# Validating the SONG-PKD Pain Instrument, a Core Outcome Measure for Pain in ADPKD

**DOI:** 10.1016/j.ekir.2024.11.015

**Published:** 2024-11-17

**Authors:** Rosanna Cazzolli, Angela Ju, Patrizia Natale, Armando Teixeira-Pinto, Martin Howell, Allison Jaure, Ronald D. Perrone, Eva Burnette, Niek F. Casteleijn, Arlene Chapman, Jonathan C. Craig, Sarah Eastty, Ron T. Gansevoort, Tess Harris, Marie C. Hogan, Shigeo Horie, Bertrand Knebelmann, Richard Lee, Karine Manera, Reem A. Mustafa, Richard Sandford, Gopala K. Rangan, Bénédicte Sautenet, Andrea K. Viecelli, Yeoungjee Cho

**Affiliations:** 1 Sydney School of Public Health, The University of Sydney, Sydney, New South Wales, Australia; 2 Centre for Kidney Research, The Children’s Hospital at Westmead, Westmead, New South Wales, Australia; 3 Department of Precision and Regenerative Medicine and Ionian Area (DIMEPRE-J), University of Bari Aldo Moro, Bari, Italy; 4 Nephrology, Dialysis and Transplantation Unit, Department of Medical and Surgical Sciences, University of Foggia, Foggia, Italy; 5 Menzies Center for Health Policy and Economics, The University of Sydney, Sydney, New South Wales, Australia; 6 Division of Nephrology, Department of Medicine, Tufts Medical Center and Tufts University School of Medicine, Boston, Massachusetts, USA; 7 United Kingdom; 8 Department of Urology, University Medical Center Groningen, University of Groningen, Groningen, The Netherlands; 9 Department of Nephrology, The University of Chicago, Chicago, Illinois, USA; 10 College of Medicine and Public Health, Flinders University, Adelaide, South Australia, Australia; 11 Department of Nephrology, University Medical Center Groningen, University of Groningen, Groningen, The Netherlands; 12 Polycystic Kidney Disease International, London, UK; 13 Division of Nephrology and Hypertension, Department of Internal Medicine, Mayo Clinic, Rochester, Minnesota, USA; 14 Department of Urology and Department of Advanced Informatics for Genetic Diseases, Juntendo University Graduate School of Medicine, Tokyo, Japan; 15 Université de Paris; APHP; Hôpital Universitaire Necker, Service de Nephrologie, Paris, France; 16 Australia; 17 Department of Internal Medicine, Division of Nephrology and Hypertension, University of Kansas Medical Center, Kansas City, Kansas, USA; 18 Academic Department of Medical Genetics, University of Cambridge, Cambridge. UK; 19 Centre for Transplant and Renal Research, Westmead Institute for Medical Research, The University of Sydney, Sydney, New South Wales, Australia; 20 Department of Renal Medicine, Westmead Hospital, Western Sydney Local Health District, Sydney, New South Wales, Australia; 21 Service de Néphrologie-Hypertension, Dialyses, Transplantation Rénale, Hôpital de Tours, Tours, France; 22 Université de Tours, Université de Nantes, INSERM, SPHERE U1246, Tours, France; 23 Australasian Kidney Trials Network, University of Queensland, Brisbane, Queensland, Australia; 24 Department of Nephrology, Princess Alexandra Hospital, Brisbane, Queensland, Australia; 25 Translational Research Institute, Brisbane, Queensland, Australia

**Keywords:** core outcome, outcome measure, pain, polycystic kidney disease, psychometric evaluation

## Abstract

**Introduction:**

Pain is a critically important outcome in autosomal dominant polycystic kidney disease (ADPKD); however, it is infrequently and inconsistently reported in clinical trials. This study aimed to validate the Standardized Outcomes in Nephrology-Polycystic Kidney Disease (SONG-PKD) Pain measure, which includes 3 items related to pain (frequency, severity, and impact on life participation) measured on a 5-point Likert scale, in adults with ADPKD.

**Methods:**

A total of 316 adults with ADPKD from 21 countries participated online. The median (interquartile range) age of participants was 56 (44–66) years, 219 (69%) were female, and 222 (70%) had a university degree or higher. Participants completed a demographic questionnaire, brief medical history, and 4 pain measures at baseline. The pain measures were readministered 2 days later. Internal consistency was evaluated with Cronbach’s alpha. Test-retest reliability was assessed using intraclass correlation coefficient (ICC), and convergent validity was assessed using Spearman’s rho. Known groups comparisons for patients with or without a history of kidney complications were performed using a Mann-Whitney rank sum test.

**Results:**

The SONG-PKD Pain measure demonstrated high internal consistency (0.94, 95% confidence interval [CI]: 0.93–0.95) and test-retest reliability (0.92, 95% CI: 0.90–0.94). There was a high convergence of SONG-PKD Pain with the Brief Pain Inventory-Short Form (BPI-SF; 0.84, 95% CI: 0.80–0.87) and a visual analog scale (VAS; 0.84, 95% CI: 0.81–0.87). There was a significant difference in the median scores of patients with and without a history of complications (4.0 vs. 0.0, *P* < 0.001).

**Conclusion:**

SONG-PKD Pain instrument is a brief and simple measure that has demonstrated strong psychometric properties.

## Graphical abstract

**Figure:**
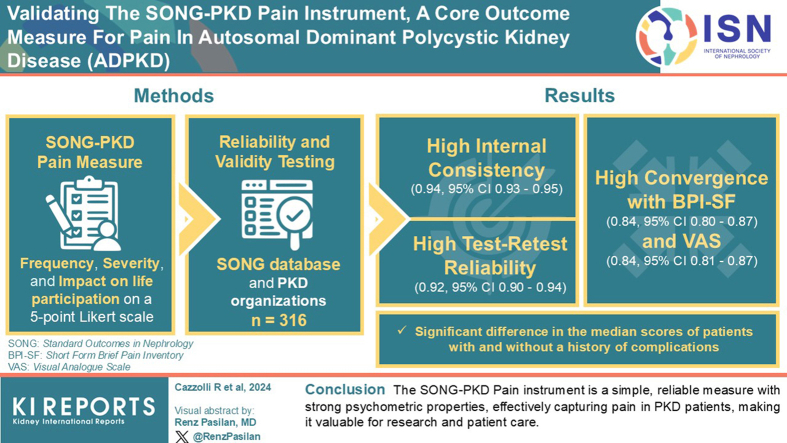

See Commentary on Page 309

Pain resulting from cyst growth and enlargement of kidneys affects more than 60% of adults with ADPKD.^1^^,^^2^ The pain associated with ADPKD can be acute, resulting from cyst-related complications such as bleeding, rupture, and infection; or chronic, caused by structural pressures on the kidney because of cyst expansion, abdominal distension, and compression of other organs; or sensitization after an acute pain-related event.^1^ The onset of pain can be severely debilitating and is associated with poor quality of life, depression, impaired sexual and social functioning, and sleep disturbance.^3^, ^4^, ^5^, ^6^, ^7^

Pain in patients with ADPKD is difficult to manage in clinical practice.^1^ Available strategies include dietary and lifestyle interventions, analgesics, and surgical intervention^8^, ^9^, ^10^, ^11^; however, 39% of patients report inadequate pain relief and dissatisfaction with care and treatment.^8^ The challenge of managing pain in clinical practice is exacerbated by the infrequent and inconsistent reporting of pain in ADPKD trials.^12^ In a review of 68 clinical trials in ADPKD, only 16 (23.5%) reported pain as an outcome.^12^ The measures used to assess ADPKD pain in trials are often not specifically developed or validated for use in people with ADPKD and vary widely in terms of the pain dimensions captured, limiting the extent to which comparisons can be made between trials. Although there have been outcome measures developed for ADPKD, which includes pain measurements, these tools are often too long to be implemented as a core outcome measure (i.e., to be implemented in all ADPKD trials), or developed for individual studies, and not validated for broader utilization.^13^, ^14^, ^15^ A validated core outcome measure for pain in ADPKD is needed to ensure consistent reporting across trials, so that research evidence can better inform clinical decision-making to manage pain and improve outcomes.

The international SONG-PKD initiative has identified pain as a core outcome for patients with ADPKD and has defined it as an outcome of critical importance to be measured and reported in all trials in patients with ADPKD.^5^ Pain is a complex and subjective experience, and is most appropriately assessed by a patient-reported outcome measure. The SONG-PKD Pain measure was developed with the input of patients, caregivers, and health professionals, in accordance with the consensus-based standards for the selection of health measurement instruments guidelines.^16^^,^^17^ The process included a systematic review of patient-reported outcome measures previously used to report pain in ADPKD trials,^18^ followed by a multistakeholder workshop to elicit preferences for the most relevant dimensions of pain, to include in a pain measure for ADPKD.^6^ Core outcome measures need to be feasibly implemented across all trials relevant to the target population.^17^ Given the lack of a validated pain measure for ADPKD, and the complexity and length of previously used measures, there was consensus on the need for a new diagnostic tool with a short completion time and ease of administration to ensure feasibility of routine and universal administration in ADPKD clinical trials.^6^ In addition, none of the previously used measures captured all the dimensions of pain considered most relevant by the workshop delegates, namely its severity, frequency, and its impact on life participation.^6^ The SONG-PKD Pain measure was developed based on these recommendations.

The consensus-based standards for the selection of health measurement instruments checklist recommends the assessment of a prescribed set of psychometric properties, to establish the validity of patient-reported outcome measures for the condition and population they are intended to measure.^19^ In this study, we aimed to examine the reliability (stability and internal consistency) and construct validity (convergent validity) properties of the SONG-PKD Pain measure, in accordance with the consensus-based standards for the selection of health measurement instruments recommendations.

## Methods

### Selection and Recruitment of Participants

Participants were eligible to participate if they were aged 18 years or older, able to read and write in English, and able to provide informed consent. Invitations were sent by email through the Standardized Outcomes in Nephrology (SONG) database. Invitations were also distributed through patient organizations (e.g., PKD Australia and PKD International). We estimated a sample size of 450 participants to allow the estimation of the ICC statistics with a 95% CI precision of 0.05, assuming an expected ICC of 0.7 and 10% of missing data in one of the observations. If the observed ICC was higher than 0.7, the CI width would be smaller. A similar precision was achieved for the Cronbach's alpha estimate, for an expected value of 0.7. Ethics approval was obtained from The University of Sydney (2022/278).

### Measures

#### The SONG-PKD Pain Measure

The SONG-PKD Pain measure includes 3 items relating to separate dimensions of pain, namely frequency, severity, and impact, which are each scored from 0 to 4 on a 5-point Likert scale. The Flesch-Kincaid Readability Grade Level of SONG-PKD Pain is Grade 4 (US),^20^ which is categorized as very easy to read. An overall score for pain was obtained by calculating the sum of the 3-item responses, resulting in a scale ranging from 0 (no pain) to 12 (highest pain score). The recall period of the SONG-PKD Pain measure is 1 week, which differs from the 24-hour recall period of the BPI-SF comparator instrument used in this study. As part of the sensitivity analysis, a modified SONG-PKD Pain measure with recall period of 24 hours was also examined. The actual and modified SONG instruments are referred to in the results section of this report as SONG-PKD Pain Week and SONG-PKD Pain 24, respectively.

##### Content Validity of the SONG-PKD Pain Measure

**Figure 1:**
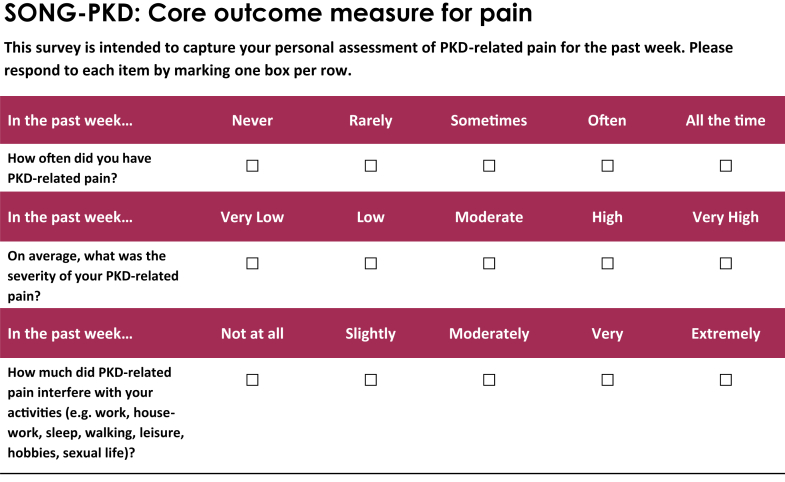
The SONG-PKD Pain instrument. SONG-PKD, Standardized Outcomes in Nephrology Polycystic Kidney Disease.

Stakeholder input and domain selection before developing the initial SONG-PKD Pain instrument established face-validity.^3^, ^4^, ^5^ We conducted cognitive interviews with patients with ADPKD to demonstrate satisfactory comprehension, retrieval of relevant cognitive information, process of judgment, and response scale usability. Interviews were conducted using Zoom videoconference from September to November 2021 with 22 patients with ADPKD (9 male, 13 female) from 5 countries (Australia, the Netherlands, Switzerland, the UK, and USA). The SONG-PKD Pain instrument (Supplementary Figure S1) was amended based on patient feedback obtained in these interviews (Sample quotations are provided in Table 1 and Supplementary Table S1). The final version of the SONG-PKD Pain instrument was reviewed by experts in patient-reported outcome development and ADPKD specialists, and is shown in Figure 1.

**Table 1:** Sample quotations from the SONG-PKD Pain instrument cognitive interviews

In the past week, how often did you have PKD-related pain?
I think what the question is asking is how many events related to pain I could count in the past week. Just asking how often have you had pain from your PKD in the last week? So that's a really straightforward one. In the past week, again, in the past 7 days. So, how often? Translating often into days or hours within days. And I'd be looking at overall time, 24-hour type, 7-day time frame for that. How often did I have kidney pain? In the past week, how often did you have PKD related pain? My first thought was ‘I wish this question was first’…My process is how often and then how bad.^a^ You'd have to make a quick judgment and say, "Well, somewhat is more than a little bit, but not as much as all the time. And quite a bit is not quite as much as all the time." It is, I think, difficult.^b^ I struggle a little bit to understand what a little bit, somewhat, quite a bit, all the time. Oh, all the time maybe's clearer, it's constant pain, right? All the rest, I'm a little bit struggling to understand how it can describe everyone's situation.^b^

In the past week, on average, what was the severity of your PKD-related pain?

Past week is fine. If you asked me about the past 2 weeks or the past month, I wouldn't have any idea. I think it's kind of, it's reasonably easy to answer, but I just take into account whether I've had any sort of particularly bad days that would bump it up. I think [the response options are] typical and fine. It's a standard Likert scale. If you build it out to a 10 point or something, it just gets too confusing I think, for people…a five point I think is quite good. I'm not sure how much the pain I'm feeling is PKD-related.^c^

In the past week, how much did PKD-related pain interfere with your activities (e.g., work, housework, sleep, walking, leisure, hobbies, sexual life)?

So, it's trying to get me to think about my whole life, not just 1 aspect of it, as in it's not just about work, factor in other life activities... And when it's talking about interfering, I would take that as did you have any effect of your pain on these? Did it stop you doing anything? So [the categories provided are] a good kind of trigger to remind you of kind of ways that it has interfered. If I had not done one of those things specifically because of the PKD pain, I'd be very well aware of that for the past week. The scale I would have the same comment, probably would have a difficult time thinking what is the difference between somewhat and quite a bit.^d^

PKD, polycystic kidney disease.

a The original order of items (severity, frequency and then impact) was changed based on feedback from the cognitive interviews.

b A new scale was chosen to reduce ambiguity in the middle responses and to better reflect the numerical nature of the question.

c An introductory statement (“This survey is intended to capture your personal assessment of PKD-related pain for the past week,” was included.

d A new scale was chosen to reduce ambiguity.

#### The BPI-SF

The BPI-SF is a widely used and validated clinical pain assessment tool^21^^,^^22^ that assesses 2 pain domains that overlap with the SONG-PKD Pain measure, severity and interference. Although the BPI-SF has not been validated in the ADPKD population, it has been tested for psychometric robustness in other chronic conditions such as cancer and osteoarthritis,^23^ and has been used in ADPKD and chronic kidney disease clinical studies.^18^^,^^24^ The recall period is 24 hours. Severity items include pain at its worst, least, and average, and now are scored from 0 (no pain) to 10 (pain as bad as you can imagine), using a numerical rating scale. BPI-SF pain severity score is calculated as a mean of the 4 item scores. Interference items assess how much pain has interfered with general activity, mood, walking, work, relations with others, sleep, and enjoyment of life, using a numerical rating scale from 0 to 10. BPI-SF pain interference score is calculated as a mean of the 7 item scores.

#### VAS

Participants were asked to give a global self-rated assessment of pain severity over the past week using a sliding VAS from 0 (no pain) to 100 (worst possible pain). The VAS is one of the most common measures of pain intensity used in clinical and research settings^25^ and evidence supports its reliability and validity diverse patient populations.^24^^,^^26^

### Data Collection

At baseline (time point 1), each participant gave informed consent and completed a demographic questionnaire, a brief medical history, and the 4 pain measures outlined in the previous section, namely SONG-PKD Pain Week, SONG-PKD Pain 24, BPI-SF, and VAS. Participants were invited to complete the pain measures again after 2 days (time point 2), and a second reminder was sent after an additional 2 days. A short time between time points 1 and 2 was chosen to minimize confounding of the data arising from significant changes in patient’s pain symptoms between administrations of the surveys.^27^ The surveys were administered online using Research Electronic Data Capture (REDCap), a secure web-based application for building and managing surveys. The survey was opened from August 2022 to November 2022.

### Data Analysis

Demographic continuous variables were described as median and interquartile range; categorical variables were summarized by counts and percentages. The scores of the different instruments were calculated as means and SD. Data analysis was conducted in SPSS Version 28.0 (IBM Corp., Armonk, NY). Psychometric properties were assessed as follows:

#### Reliability

##### Test-Retest Stability

The reproducibility of scores between time points 1 and 2 was assessed with ICC, with values ≥ 0.75 considered to indicate good to excellent reproducibility.^16^^,^^28^

##### Internal Consistency

The correlation of individual item scores within the same instrument was assessed using Cronbach’s alpha in a complete case analysis at time point 1, where values ≥ 0.70 indicated adequate consistency.^16^^,^^27^

#### Construct Validity

##### Convergent Validity

Domain level and item level convergent validity (the degree to which the scores of one instrument correlate with the scores of other instruments intended to measure the same construct) was assessed with Spearman’s rho using a complete case analysis of pain scores at time point 1. Values greater than 0.7 indicated high correlation, 0.3 to 0.7 indicated moderate correlation, and values less than 0.3 indicated low correlation.^17^^,^^19^^,^^29^ A subgroup analyses of convergence, using only data from participants who scored higher than 0 for all the measures at time point 1, was also performed.

##### Known Groups Comparison

Pain in the ADPKD patient population is unpredictable, multifaceted, and complex, making it difficult to identify definitive known groups *a priori* for comparison. It was hypothesized that patients with a history of renal complications (e.g. cyst infection, rupture, or hemorrhage) would have higher pain scores, and so this variable was used to compare groups at baseline. In an additional subgroup analysis of patients with a history of complications, “recent complications” (i.e., participants who experienced a renal complication within 4 weeks of taking the baseline survey) was selected as grouping variable. Mann-Whitney tests were used to compare scores from these prespecified groups and subgroups.

##### Acceptability

The level of data quality was assessed by data completeness and score distributions. Less than 5% of missing data for instrument score, and floor and ceiling effects < 10%, were used as criteria for acceptability.

## Results

In total, 316 people completed the baseline survey (time point 1) and 267 (84%) completed the follow-up survey (time point 2). Participants were from 21 countries, including USA (*n* = 170, 54%), UK (*n* = 49, 16%), and Australia (*n* = 44, 14%). The median (interquartile range) age of participants was 56 (44–66) years. A high proportion of the respondents were female (*n* = 219, 69%) and had completed a university degree or higher (*n* = 222, 70%). Approximately 50% of participants (*n* = 160) reported history of cyst-related complications; 20 participants (6%) reported that their most recent kidney-related complication had occurred within 4 weeks of taking the baseline survey. Other demographic details and medical characteristics are provided in Table 2.

**Table 2:** Demographic and clinical characteristics of the patient cohort

Characteristics	Baseline *n* = 316	Follow-up *n* = 267
Sex, *n* (%)		
Male	95 (30)	78 (29)
Female	219 (69)	189 (71)
Not specified	2 (1)	0 (0)
Age, yrs		
Median (IQR)	56 (44, 66)	56 (45, 67)
Ethnicity, *n* (%)		
Caucasian/European/White	288 (91)	246 (92)
Asian	11 (3)	7 (3)
Hispanic/Latin American	9 (3)	9 (3)
Other/Prefer not to say	8 (3)	5 (2)
Country, *n* (%)		
USA	170 (54)	145 (54)
UK	49 (16)	43 (16)
Australia	44 (14)	39 (15)
The Netherlands	22 (7)	19 (7)
Other^a^	31 (10)	21 (8)
Highest level of education, *n* (%)		
Primary/elementary school	1 (0)	0 (0)
High/secondary school up to year 10	9 (3)	9 (3)
High/secondary school up to year 12	24 (8)	22 (8)
Professional certificate, vocational training	57 (18)	42 (16)
University degree or higher	222 (70)	192 (72)
Other/Prefer not to say	3 (1)	2 (1)
Yrs since diagnosis, *n* (%)		
0–5	5 (2)	4 (1)
6–10	10 (3)	5 (2)
11–20	72 (23)	56 (21)
21–30	84 (27)	73 (27)
>30	141 (45)	126 (47)
Not specified	4 (1)	3 (1)
Kidney replacement therapy (*n*, %)		
Dialysis	23 (7)	20 (7)
Kidney transplantation	57 (18)	49 (18)
Cyst manifestation, *n* (%)		
Kidney	314 (99)	266 (100)
Liver	220 (70)	189 (71)
Previous cyst complications (*n*, %)	160 (51)	133 (50)
Previous medical intervention for cyst complications (*n*, %)	36 (11)	28 (10)
Nephrectomy^b^	7 (2)	5 (2)
Removal of cysts	13 (4)	10 (4)
Injection of steroid/other medication	2 (1)	1 (0.4)
Other	14 (4)	12 (4)
Nephrectomy^c^, *n* (%)		
Single	16 (5)	11 (4)
Double	9 (3)	8 (3)

IQR, interquartile range.

a Other countries at baseline: Belgium (*n* = 1), Canada (*n* = 4), China (*n* = 1), Denmark (*n* = 1), Georgia (*n* = 1), Hong Kong (*n* = 3), India (*n* = 2), Israel (*n* = 1), Italy (*n* = 3), Mexico (*n* = 1), New Zealand (*n* = 2), Nigeria (*n* = 1), Pakistan (*n* = 1), Qatar (*n* = 1), South Africa (*n* = 1), Spain (*n* = 1), Switzerland (*n* = 2); not specified (*n* = 4).

b Indicates number of participants who had a nephrectomy to manage cyst complications.

c Indicates number of participants who had a nephrectomy for any reason.

### Acceptability

**Figure 2:**
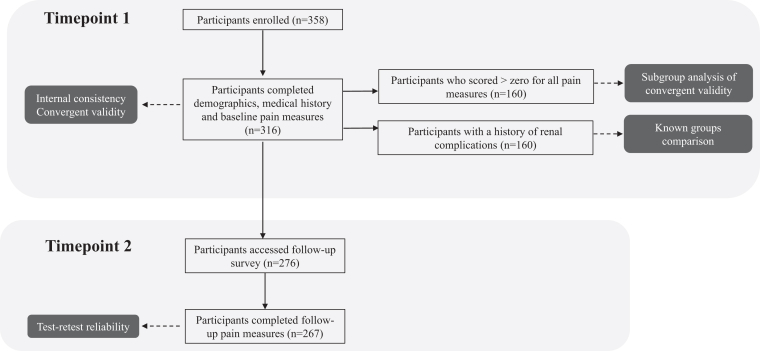
Flowchart of participants.

At baseline, 358 participants accessed the study and 88% (*n* = 316) completed all the measures. At follow-up, 276 participants (87% of those who completed the baseline questionnaires) accessed the study and 97% (*n* = 267) completed all the measures (Figure 2). The SONG-PKD Pain Week measure had a 100% completion rate at time point 1, and 99% completion rate for participants that returned at time point 2. There was no significant difference, at time point 1, in the median SONG-PKD Week scores of participants who did or did not return to complete the measures at time point 2 (2.0 vs. 2.0 *P* = 0.9). The reasons for missing data could not be ascertained because the survey was administered online. Participants with missing data were removed before data analysis.

The floor effect (percentage of patients with the lowest possible score, i.e., score = 0) for the SONG-PKD Pain Week instrument was 40% and the ceiling effect (percentage of patients with the highest possible score, i.e., score = 12) was 0.3% (Table 3). Floor effects for the SONG-PKD Pain 24 instrument (44%), BPI-SF severity (34%), BPI-SF interference (40%), and VAS (27%) were also high (Table 3). Similar results were observed at time point 2 (Supplementary Table S2). Distribution of item and instrument scores for the SONG-PKD Pain Week measure at time point 1 are shown in Supplementary Figures S2 and S3, respectively.

**Table 3:** Mean scores, internal consistency (Cronbach’s alpha), and test-retest reliability (ICC) for each of the instruments used in this study

Instrument	Mean Score^a^ (SD)	Floor^b^ (%)	Ceiling^b^ (%)	Skewness	Cronbach’s alpha (95% CI)	ICC^c^ (95% CI)
SONG-PKD Pain Week	3.0 (3.3)	40.2	0.3	0.71	0.94 (0.93–0.95)	0.92 (0.90–0.94)
*Comparator Instruments:*						
SONG-PKD Pain 24	2.8 (3.3)	44.0	0.6	0.86	0.93 (0.92–0.95)	0.85 (0.82–0.88)
BPI-SF Severity	1.8 (2.2)	34.2	0.3	1.28	0.94 (0.93–0.95)	0.87 (0.84–0.90)
BPI-SF Interference	2.0 (2.6)	39.6	0.6	1.20	0.97 (0.97–0.98)	0.86 (0.83–0.89)
VAS	22.2 (26.2)	27.2	0.3	1.03	n/a	0.92 (0.90–0.93)

BPI-SF, brief pain inventory-short form; CI, confidence interval; ICC, intraclass correlation coefficient; n/a, not applicable; SONG-PKD, Standardized Outcomes in Nephrology-Polycystic Kidney Disease; VAS, visual analog scale.

a Mean scores as calculated in a complete case analysis of baseline data (*n* = 316).

b Percent frequency of minimum (floor)/maximum (ceiling) scores in a complete case analysis of baseline data (*n* = 316).

c ICC for scores at time points 1 and 2, as calculated by a complete case analysis of follow-up data (*n* = 267).

### Reliability

Cronbach’s alpha for the SONG-PKD Pain Week instrument at time point 1 was 0.94 (95% CI: 0.93–0.95), indicating a high level of internal consistency (Table 3). SONG-PKD Pain Week performed similarly for males (*n* = 95, 0.94, 95% CI: 0.92–0.96) and females (*n* = 219, 0.94, 95% CI: 0.92–0.95). Other instruments used in the study also had high levels of internal consistency (Table 3). The test-retest analysis demonstrated that the SONG-PKD Pain Week scores had a high level of stability between time points 1 and 2, with an ICC of 0.92 (95% CI: 0.90–0.94; Table 3). ICC of the SONG-PKD Pain Week instrument for males was 0.87 (*n* = 78, 95% CI: 0.80–0.91) and for females was 0.94 (*n* = 189, 95% CI: 0.92–0.95). The SONG-PKD Pain 24, BPI-SF severity, BPI-SF interference, and VAS scores had similarly high levels of stability with ICCs of 0.85 (95% CI: 0.82–0.88), 0.87 (95% CI: 0.84–0.90), 0.86 (95% CI: 0.83–0.89), and 0.92 (95% CI: 0.90–0.93), respectively (Table 3).

### Construct Validity

The SONG-PKD Pain Week instrument demonstrated high domain-level convergent validity with the BPF-SF severity domain (Spearman’s rho: 0.84, 95% CI: 0.80–0.87), the BPI-SF interference domain (Spearman’s rho: 0.81, 95% CI: 0.76–0.84), and the VAS (Spearman’s rho: 0.84, 95% CI: 0.81–0.87). The SONG-PKD Pain 24 instrument measure highly correlated with the SONG-PKD Pain Week instrument measure (Spearman’s rho: 0.95, 95% CI: 0.93–0.96) and demonstrated comparably high convergence with the other instruments’ measures used in this study (Table 4). Because the BPI-SF does not contain a frequency item, we also explored item-level convergence. The SONG-PKD Pain Week severity and impact items independently demonstrated convergence with the severity and interference scales of the BPI-SF measure, and with the VAS (Supplementary Table S3).

**Table 4:** Convergent validity^a^ between instruments as calculated in a complete case analysis of baseline data (*n* = 316)

	SONG-PKD Pain 24	BPI-SF Severity	BPI-SF Interference	VAS
SONG-PKD Pain Week *Comparator instruments:*	0.95 (0.93–0.96)	0.84 (0.80–0.87)	0.81 (0.76–0.84)	0.84 (0.81–0.87)
SONG-PKD Pain 24	-	0.85 (0.82–0.88)	0.82 (0.77–0.85)	0.82 (0.78–0.85)
BPI-SF Severity	-	-	0.86 (0.82–0.88)	0.87 (0.84–0.90)
BPI-SF Interference	-	-	-	0.86 (0.83–0.89)

BPI-SF, brief pain inventory-short form; SONG-PKD, Standardized Outcomes in Nephrology-Polycystic Kidney Disease; VAS, visual analog scale.

a Convergent validity is reported as Spearman’s rho correlation coefficient; the 95% confidence interval is in parentheses.

Due to high floor effects and the moderate to high skewness in the distribution of scores observed for all measures used in this study, a subgroup analysis of convergence was performed using only data from participants who scored >0 for all the instruments at time point 1 (*n* = 160). The floor and ceiling effects of this data set fell within the acceptable range, and internal consistency for each of the measures was still high (Table 5). Convergent validity of the SONG-PKD Pain Week measure was maintained, as demonstrated by high correlation coefficients with the comparator measures of pain (Table 6).

**Table 5:** Mean scores, floor/ceiling effects, and internal consistency (Cronbach’s alpha) at baseline for participants scoring higher than 0 for all measures used in the study (*n* = 160)

Instrument	Mean Score (SD)	Floor^a^ (%)	Ceiling^a^ (%)	Skewness	Cronbach’s Alpha (95% CI)
SONG PKD Pain Week	5.64 (2.55)	4.4	0.6	0.12	0.85 (0.81–0.89)
*Comparator Instruments:*					
SONG-PKD Pain 24	5.41 (2.72)	6.9	1.3	0.22	0.85 (0.81–0.89)
BPI-SF Severity	3.16 (2.09)	1.9	0.6	0.62	0.93 (0.91–0.95)
BPI-SF Interference	3.47 (2.58)	5.6	0.6	0.38	0.96 (0.95–0.97)
VAS	38.74 (25.60)	0.6	0.6	0.30	n/a

BPI-SF, brief pain inventory-short form; CI, confidence interval; n/a, not applicable; SONG-PKD, Standardized Outcomes in Nephrology-Polycystic Kidney Disease; VAS, visual analog scale.

a Percent frequency of minimum (floor)/maximum (ceiling) scores.

**Table 6:** Convergent validity^a^ including only those participants who scored higher than 0 for all measures at baseline (*n* = 160)

	SONG-PKD Pain 24	BPI Severity	BPI Interference	VAS
SONG-PKD Pain Week	0.90 (0.86–0.93)	0.76 (0.69–0.82)	0.75 (0.68–0.82)	0.78 (0.70–0.83)
*Comparator Instruments:*				
SONG-PKD Pain 24		0.79 (0.72–0.84)	0.78 (0.71–0.84)	0.76 (0.68–0.82)
BPI-SF Severity	-	-	0.80 (0.73–0.85)	0.83 (0.77–0.87)
BPI-SF Interference	-	-	-	0.79 (0.72–0.84)

BPI-SF, brief pain inventory-short form; SONG-PKD, Standardized Outcomes in Nephrology-Polycystic Kidney Disease; VAS = visual analog scale.

a Convergent validity reported as Spearman’s rho correlation coefficient; the 95% confidence interval is in parentheses.

### Known Groups Comparison

SONG-PKD Pain Week scores for participants with a history of kidney-related complications (*n* = 160) were significantly higher than those without previous complications (*n* = 155) (medians: 4.0 vs. 0.0, *P <* 0.001). Similarly, for patients with a history of complications, SONG-PKD Pain Week scores were significantly higher for those who had experienced a complication in the previous 4 weeks (*n* = 20) compared to those who had not (*n* = 140; medians: 6.5 vs. 4.0, *P* = 0.01).

## Discussion

Pain has been identified by patients, caregivers, and health professionals as a critically important core outcome to be reported in trials in patients with ADPKD.^5^ The SONG-PKD Pain instrument reflects those aspects of pain that are of highest priority to patients, namely severity, frequency, and impact on life participation, and was developed using a comprehensive consensus-based process involving patients with ADPKD, health professionals and researchers.^3^, ^4^, ^5^ This report describes a psychometric evaluation of the SONG-PKD Pain measure, which was found to satisfy reliability and validity criteria in accordance with the recommended US Food and Drug Administration and consensus-based standards for the selection of health measurement instruments guidelines.^16^^,^^30^

SONG-PKD Pain measure demonstrated acceptable internal consistency, test-retest reliability, and high item-level and domain-level convergence with the BPI-SF and VAS. The high floor effects observed for all instruments used in this study is consistent with those observed in previous studies assessing pain in the ADPKD population,^14^ and suggests that pain may not have been problematic for many participants at the time of filling out the surveys. High floor and ceiling effects may also indicate insensitivity of the measure to the occurrence of pain. However, if so, this is true for all the instruments used in the study. High ceiling and floor effects also limit the range and variability of gathered data and may introduce bias in statistical analysis on correlations. However, our subgroup analyses by excluding patients who scored 0 for any of the pain measures, which minimized floor and ceiling effects, demonstrated that the strong psychometric properties of the SONG-PKD Pain measure were retained. Taken together, the results demonstrate that the SONG-PKD Pain measure seems to be a robust measure of PKD-related pain.

Other patient-reported outcome measures that include pain items have been validated for use in patients with ADPKD. The polycystic liver disease questionnaire^13^ is not specific to ADPKD and does not assess the impact on life participation, which is the most important pain domain for patients.^3^^,^^4^ The ADPKD impact scale^15^ includes 3 items related to pain and its effect on daily living but does not capture severity and frequency. The ADPKD-specific pain assessment tool^31^ includes severity, impact, and frequency domains. The tool is comprehensive and ADPKD-specific; however, its length and complexity preclude its feasibility as a core outcome measure to be included for all future ADPKD trials. At the time of conducting this study, the ADPKD pain and discomfort scale was in development. This instrument, which distinguishes between dull pain, sharp pain, and a feeling of fullness, and includes frequency, severity, and interference domains, has since been validated^14^, and is a promising tool for use in trials where pain is a primary outcome, and a more comprehensive understanding of pain patterns is required. SONG-PKD Pain is the only ADPKD-specific pain measurement tool with the brevity and ease of completion to make it feasible to use in all ADPKD trials, including trials where pain is not a main research focus.

The SONG-PKD Pain instrument was developed to evaluate ADPKD-related pain over a period of 1 week. Although the ideal reporting period for pain assessment is debated,^32^ recalled average pain over 1 week is considered a reliable and valid measure of pain intensity^29^^,^^33^^,^^34^ and was chosen for our instrument based on the expressed preferences of patients. We acknowledge that the relatively short recall period may be more conducive to assessing chronic pain; whether the SONG-PKD Pain instrument is able to capture changes in episodic or acute pain symptoms will need further analysis in a longitudinal study with multiple time points of administration.

There are some limitations to this study, including the generalizability of the results to populations not captured within the study cohort. The surveys were administered in English and online, which excluded the participation of non-English speakers and those without access to online platforms or with low digital literacy. Most of the participants were from high income countries and had high educational attainment. Further work is needed to assess the psychometric robustness of the measure in other populations, and to determine cultural and language validity. Majority of data were obtained from patients with > 20 years of known diagnosis of ADPKD, which may limit applicability of findings to those who are newly diagnosed. Despite not having achieved the planned sample size, the precision of the estimated statistics was maintained or even improved because of the point estimates being considerable higher than anticipated in the sample size calculation. The validity and reliability assessments were not affected by the high proportion of females in the sample population, because the measures performed equally well whether completed by male or female participants. Our exploratory known-group analyses suggests that the SONG-PKD Pain instrument has the potential to distinguish between patients with and without a history of cyst-related complications, including patients with a very recent complication. However, further work is required to conclusively establish known-group validity for this measure. Other psychometric properties such as responsiveness and minimal clinically significant difference are also yet to be determined.

The SONG-PKD Pain Instrument is designed to be used in clinical trials. However, the instrument may be potentially used in clinical settings, for example to screen for and prompt discussion about pain, or to facilitate decision-making. This initial evidence to support validity and reliability of the SONG-PKD Pain instrument indicated that it is an appropriate patient-reported outcome measure for patients with ADPKD. The use of the instrument across trials of ADPKD can provide a standardized measure of pain that is relevant and meaningful to patients. This will contribute to an evidence-base in which patients, caregivers and health professionals can compare and consider the effect of interventions on pain for better decision-making and outcomes for patients.

## Disclosure

RDP is a consultant to Otsuka and has been an advisor to Regulus, Vertex, Retex, and Sanofi-Genzyme; funds were paid to institution. AKV has received speaker’s honoraria from CSL Seqirus and Alexion. All the other authors declared no competing interests.

## Supplementary Material

Supplementary File (PDF) **Figure S1.** Initial SONG-PKD Pain Instrument. **Figure S2.** Frequency of SONG-PKD Pain Week item scores at baseline. **Figure S3.** Frequency of SONG-PKD Pain Week total scores at baseline. **Table S1.** Illustrative quotes from the cognitive interviews. **Table S2.** Mean scores, floor/ceiling effects, and internal consistency (Cronbach’s alpha) at follow-up. **Table S4.** COSMIN Reporting guideline for studies on measurement properties of patient-reported outcome measures. **Table S3.** Item-specific correlations as calculated in a complete case analysis of baseline data.

## Footnotes

Supplementary File (PDF)
**Figure S1.** Initial SONG-PKD Pain Instrument. **Figure S2**. Frequency of SONG-PKD Pain Week item scores at baseline. **Figure S3.** Frequency of SONG-PKD Pain Week total scores at baseline. **Table S1**. Illustrative quotes from the cognitive interviews. **Table S2.** Mean scores, floor/ceiling effects, and internal consistency (Cronbach’s alpha) at follow-up. **Table S4**. COSMIN Reporting guideline for studies on measurement properties of patient-reported outcome measures. **Table S3.** Item-specific correlations as calculated in a complete case analysis of baseline data.

## References

[1] Bajwa Z.H., Sial K.A., Malik A.B., Steinman T.I. (2004). Pain patterns in patients with polycystic kidney disease. Kidney Int.

[2] Grantham J.J., Chapman A.B., Torres V.E. (2006). Volume progression in autosomal dominant polycystic kidney disease: the major factor determining clinical outcomes. Clin J Am Soc Nephrol.

[3] Cho Y., Rangan G., Logeman C. (2020). Core outcome domains for trials in autosomal dominant polycystic kidney disease: an international Delphi survey. Am J Kidney Dis.

[4] Cho Y., Sautenet B., Gutman T. (2019). Identifying patient-important outcomes in polycystic kidney disease: an international nominal group technique study. Nephrol (Carlton).

[5] Cho Y., Tong A., Craig J.C. (2021). Establishing a core outcome set for autosomal dominant polycystic kidney disease: report of the standardized outcomes in nephrology-polycystic kidney disease (SONG-PKD) consensus workshop. Am J Kidney Dis.

[6] Natale P., Perrone R.D., Tong A. (2022). Establishing a core outcome measure for pain in patients with autosomal dominant polycystic kidney disease: a consensus workshop report. Clin Kidney J.

[7] Tong A., Rangan G.K., Ruospo M. (2015). A painful inheritance-patient perspectives on living with polycystic kidney disease: thematic synthesis of qualitative research. Nephrol Dial Transplant.

[8] Tellman M.W., Bahler C.D., Shumate A.M., Bacallao R.L., Sundaram C.P. (2015). Management of pain in autosomal dominant polycystic kidney disease and anatomy of renal innervation. J Urol.

[9] van Luijk F., Gansevoort R.T., Blokzijl H. (2023). Multidisciplinary management of chronic refractory pain in autosomal dominant polycystic kidney disease. Nephrol Dial Transplant.

[10] Savige J., Tunnicliffe D.J., Rangan G.K. (2015). KHA-CARI autosomal dominant kidney disease guideline: management of chronic pain. Semin Nephrol.

[11] Casteleijn N.F., Visser F.W., Drenth J.P. (2014). A stepwise approach for effective management of chronic pain in autosomal-dominant polycystic kidney disease. Nephrol Dial Transplant.

[12] Sautenet B., Cho Y., Gutman T. (2020). Range and variability of outcomes reported in randomized trials conducted in patients with polycystic kidney disease: a systematic review. Am J Kidney Dis.

[13] Neijenhuis M.K., Gevers T.J., Hogan M.C. (2016). Development and validation of a disease-specific questionnaire to assess patient-reported symptoms in polycystic liver disease. Hepatology.

[14] Oberdhan D., Cole J.C., Atkinson M.J., Krasa H.B., Davison S.N., Perrone R.D. (2023). Development of a patient-reported outcomes tool to assess pain and discomfort in autosomal dominant polycystic kidney disease. Clin J Am Soc Nephrol.

[15] Oberdhan D., Cole J.C., Krasa H.B. (2018). Development of the autosomal dominant polycystic kidney disease impact scale: a new health-related quality-of-life instrument. Am J Kidney Dis.

[16] Mokkink L.B., Terwee C.B., Patrick D.L. (2010). The COSMIN checklist for assessing the methodological quality of studies on measurement properties of health status measurement instruments: an international Delphi study. Qual Life Res.

[17] Prinsen C.A., Vohra S., Rose M.R. (2016). How to select outcome measurement instruments for outcomes included in a “Core Outcome Set” - a practical guideline. Trials.

[18] Natale P., Hannan E., Sautenet B. (2021). Patient-reported outcome measures for pain in autosomal dominant polycystic kidney disease: a systematic review. PLoS One.

[19] Gagnier J.J., Lai J., Mokkink L.B., Terwee C.B. (2021). COSMIN reporting guideline for studies on measurement properties of patient-reported outcome measures. Qual Life Res.

[20] Kincaid J.P.F., Robert P., Rogers R.L., Chissom B.S. Derivation of new readability formulas (automated readability index, fog count and Flesch reading ease formula) for navy enlisted personnel (1975). University of Central Florida, Institute for Simulation and Training. https://stars.library.ucf.edu/istlibrary/56.

[21] Cleeland C.S., Ryan K.M. (1994). Pain assessment: global use of the Brief Pain Inventory. Ann Acad Med Singap.

[22] Keller S., Bann C.M., Dodd S.L., Schein J., Mendoza T.R., Cleeland C.S. (2004). Validity of the brief pain inventory for use in documenting the outcomes of patients with noncancer pain. Clin J Pain.

[23] Cleeland C.S. (Published 2009). The brief pain inventory: user guide. https://www.mdanderson.org/content/dam/mdanderson/documents/Departments-and-Divisions/Symptom-Research/BPI_UserGuide.pdf.

[24] Davison S.N., Rathwell S., Ghosh S., George C., Pfister T., Dennett L. (2021). The prevalence and severity of chronic pain in patients with chronic kidney disease: a systematic review and meta-analysis. Can J Kidney Health Dis.

[25] Ferreira-Valente M.A., Pais-Ribeiro J.L., Jensen M.P. (2011). Validity of four pain intensity rating scales. Pain.

[26] Upadhyay C., Cameron K., Murphy L., Battistella M. (2014). Measuring pain in patients undergoing hemodialysis: a review of pain assessment tools. Clin Kidney J.

[27] Terwee C.B., Bot S.D., de Boer M.R. (2007). Quality criteria were proposed for measurement properties of health status questionnaires. J Clin Epidemiol.

[28] Koo T.K., Li M.Y. (2016). A guideline of selecting and reporting intraclass correlation coefficients for reliability research. J Chiropr Med.

[29] Frost M.H., Reeve B.B., Liepa A.M., Stauffer J.W., Hays R.D., FDAP-ROCMG M. (2007). What is sufficient evidence for the reliability and validity of patient-reported outcome measures?. Value Health.

[30] Patient-reported outcome measures: use in medical product development to support labeling claims. United States Food and Drug Administration. https://www.fda.gov/media/77832/download.

[31] El-Damanawi R., Lee M., Harris T. (2021). Developing a patient-centred tool for pain measurement and evaluation in autosomal dominant polycystic kidney disease. Clin Kidney J.

[32] Norquist J.M., Girman C., Fehnel S., DeMuro-Mercon C., Santanello N. (2012). Choice of recall period for patient-reported outcome (PRO) measures: criteria for consideration. Qual Life Res.

[33] Broderick J.E., Schwartz J.E., Vikingstad G., Pribbernow M., Grossman S., Stone A.A. (2008). The accuracy of pain and fatigue items across different reporting periods. Pain.

[34] Jamison R.N., Raymond S.A., Slawsby E.A., McHugo G.J., Baird J.C. (2006). Pain assessment in patients with low back pain: comparison of weekly recall and momentary electronic data. J Pain.

