# Supplementary material for: Validating the SONG-PKD Pain Instrument, a Core Outcome Measure for Pain in ADPKD
Source: Kidney Int Rep. 2024 Nov 17;10(2):447–56. doi: 10.1016/j.ekir.2024.11.015 (PMC11843306; doi:10.1016/j.ekir.2024.11.015)
Supplement: Supplementary File (PDF) — Figure S1. Initial SONG-PKD Pain Instrument. Figure S2. Frequency of SONG-PKD Pain Week item scores at baseline. Figure S3. Frequency of SONG-PKD Pain Week total scores at baseline. Table S1. Illustrative quotes from the cognitive interviews. Table S2. Mean scores, floor/ceiling effects, and internal consistency (Cronbach’s alpha) at follow-up. Table S4. COSMIN Reporting guideline for studies on measurement properties of patient-reported outcome measures. Table S3. Item-specific correlations as calculated in a complete case analysis of baseline data. [file mmc1.pdf]

SUPPLEMENTARY MATERIAL

Contents

Figure S1	Initial SONG-PKD Pain Instrument
Table S1	Illustrative quotes from the cognitive interviews
Table S2	Mean scores, floor/ceiling effects and internal consistency (Cronbach's alpha) at follow-up
Table S3	Item-specific correlations as calculated in a complete case analysis of baseline data
Figure S2	Frequency of SONG-PKD Pain Week item scores at baseline
Figure S3	Frequency of SONG-PKD Pain Week total scores at baseline
Table S4	COSMIN Reporting guideline for studies on measurement properties of patient reported outcome measures

Figure S1: Initial SONG-PKD Pain Instrument: The SONG-PKD Pain instrument was developed to include pain dimensions identified by patients, caregivers and health professionals to be the most important to capture in ADPKD clinical trials.

In the past week…	None	Low	Moderate	High	Very high
On average, what was the severity of your PKD-related pain?	☐	☐	☐	☐	☐
**In the past week…**	**Not at all**	**A little bit**	**Somewhat**	**Quite a bit**	**All the time**
How often did you have PKD-related pain?	☐	☐	☐	☐	☐
How much did PKD-related pain interfere with your activities (e.g. work, housework, sleep, walking, leisure, hobbies, sexual life)?	☐	☐	☐	☐	☐

Table S1 Illustrative quotes from the cognitive interviews: To establish content validity, we assessed the SONG-PKD Pain Instrument for comprehension, retrieval of relevant cognitive information, processes of judgement and response scale usability, in a series of cognitive interviews with 22 patients.

Domain	Illustrative Quotes
**Q1 Comprehension**	Well what confuses me the most is I didn't read the bit in the past week. So I think I would've preferred that in the question<br>On average, the median point, if I have high pains one day, low pain another day, the average would be middle.<br>Severity of pain in relation to PKD. So specifically PKD with kidneys
**Q1 Retrieval**	Just recall the last week, just memory<br>Past week is fine. If you asked me about the past two weeks or the past month, I wouldn't have any idea.<br>So I'm trying to estimate an average and I'm thinking about it purely related to my kidneys.
**Q1 Judgement**	Sometimes it's difficult to judge, I must admit, because pain is there, but you might take it for granted, so you wouldn't really answer that it was a week with pain<br>I think it's kind of, it's reasonably easy to answer, but I just take into account whether I've had any sort of particularly bad days that would bump it up.<br>I'm not sure how much the pain I'm feeling is PKD related<br>Not ‘how often’, because it kind of implies that it comes in fits and farts
**Q1 Response**	I think [the response options are] typical and fine<br>It's a standard Likert scale. If you build it out to a 10 point or something, it just gets too confusing I think, for people…a five point I think is quite good.<br>For me, very high is easy to understand. Low is like a background thing. But moderate and high, yeah, what do I ascribe to that?
**Q2 Comprehension**	I think what the question is asking is how many events related to pain I could count in the past week.<br>Just asking how often have you had pain from your PKD in the last week? So that's a really straightforward one
**Q2 Retrieval**	In the past week, again, in the past seven days. So, how often? Translating often into days or hours within days. And I'd be looking at overall time, 24 hour type, seven day time frame for that. How often did I have kidney pain?<br>In the past week, how often did you have PKD related pain? My first thought was ‘I wish this question was first’…My process is how often and then how bad.
**Q2 Judgement**	I'd be a little bit kind of grey as to, well, should I put in four days worth as somewhat? Should I put in six days worth as quite a bit?<br>You'd have to make a quick judgment and say, "Well, somewhat is more than a little bit, but not as much as all the time. And quite a bit is not quite as much as all the time,". It is, I think, difficult.<br>Again, it's the past week versus an average week for me, which is if I couldn't clarify, I'd be tempted to revert to an average week.
**Q2 Response**	How often did you have it and the scale don't quite jive, until you get to quite a bit and all the time. How often did you have it? ‘Somewhat’ doesn't really to me sound like ‘how often’?<br>I struggle a little bit to understand what a little bit, somewhat, quite a bit, all the time. Oh, all the time maybe's clearer, it's constant pain, right? All the rest, I'm a little bit struggling<br>I'd probably end up ignoring this somewhat in the middle because it was ambiguous to me. I mean, in English, what is the difference between quite a bit and somewhat?
**Q3 Comprehension**	So how much did the pain sort of interrupt your life, whether it's … so much that you couldn't do certain things that you'd want to be able to do<br>Whether the pain that I have from my PKD, in the last week, kept me from doing something that I should do or wanted to do<br>So, it's trying to get me to think about my whole life, not just one aspect of it, as in it's not just about work, factor in other life activities... And when it's talking

Domain	Illustrative Quotes
	about interfering, I would take that as did you have any effect of your pain on these? Did it stop you doing anything?
**Q3 Retrieval**	I'd probably look at the different kind of classifications, work, house work and think, how much was I interrupted in doing this and then kind of balance it out<br>I think a week here is a good amount of time because I'm not sure we remember exactly what we were doing eight days ago. So I think this is about the right frame of time as well<br>If I had not done one of those things specifically because of the PKD pain, I'd be very well aware of that for the past week
**Q3 Judgement**	If you just ask, oh, how much did it interfere? I might go, oh, it wasn't too bad. And then when I look at work, housework, I'd actually think, well, no, I couldn't stand to do the washing up the other day. I had to lay down in my lunch break and things like that.<br>So [the categories provided are] a good kind of trigger to remind you of kind of ways that it has interfered
**Q3 Response**	The scale I would have the same comment, probably would have a difficult time thinking what is the difference between somewhat and quite a bit

Table S2. Mean scores, floor/ceiling effects and internal consistency (Cronbach's alpha) at follow-up (n=267)

	Mean Score[a] (SD)	Floor[b] (%)	Ceiling[b] (%)	Skewness	Cronbach's alpha[a] (95% CI)
**SONG-PKD Pain 24**	2.6 (3.3)	50.6	0.7	0.95	0.94 (0.93 to 0.96)
**SONG-PKD Pain Week**	2.9 (3.2)	41.2	0.7	0.76	0.95 (0.93 to 0.96)
**BPI-SF Severity**	1.8 (2.3)	39.3	0.4	1.14	0.95 (0.94 to 0.96)
**BPI-SF Interference**	1.8 (2.5)	46.1	0.7	1.21	0.98 (0.97 to 0.98)
**VAS**	22.7 (27.3)	30.0	0.7	0.99	-

a: Mean scores and Cronbach's alpha as calculated in a complete case analysis of follow-up data (n=267)
b: Percent frequency of minimum (floor)/maximum (ceiling) scores in a complete case analysis of follow-up data (n=267)
Abbreviations: BPI-SF = brief pain inventory short form; CI = confidence interval; SD = standard deviation; SONG-PKD = standardized outcomes in nephrology-polycystic kidney disease; VAS = visual analogue scale

Table S3 Item-specific correlations as calculated in a complete case analysis of baseline data (n=316)

	Item	BPI Severity	BPI Interference	VAS
**SONG-PKD Pain 24**	Severity Item	0.83 (0.79 to 0.86)	0.78 (0.74 to 0.82)	0.80 (0.76 to 0.84)
	Impact Item	0.77 (0.72 to 0.81)	0.78 (0.74 to 0.82)	0.75 (0.70 to 0.80)
**SONG-PKD Pain Week**	Severity Item	0.82 (0.78 to 0.85)	0.79 (0.74 to 0.83)	0.83 (0.79 to 0.86)
	Impact Item	0.76 (0.71 to 0.80)	0.79 (0.74 to 0.83)	0.78 (0.73 to 0.82)

a: Convergent validity is reported as Spearman's rho correlation coefficient; the 95% confidence interval is in parentheses 95% CI, 2-tailed; Estimation is based on Fischer's r-to-z transformation; Estimation of standard error is based on the formula proposed by Fieller, Hartley, and Pearson.

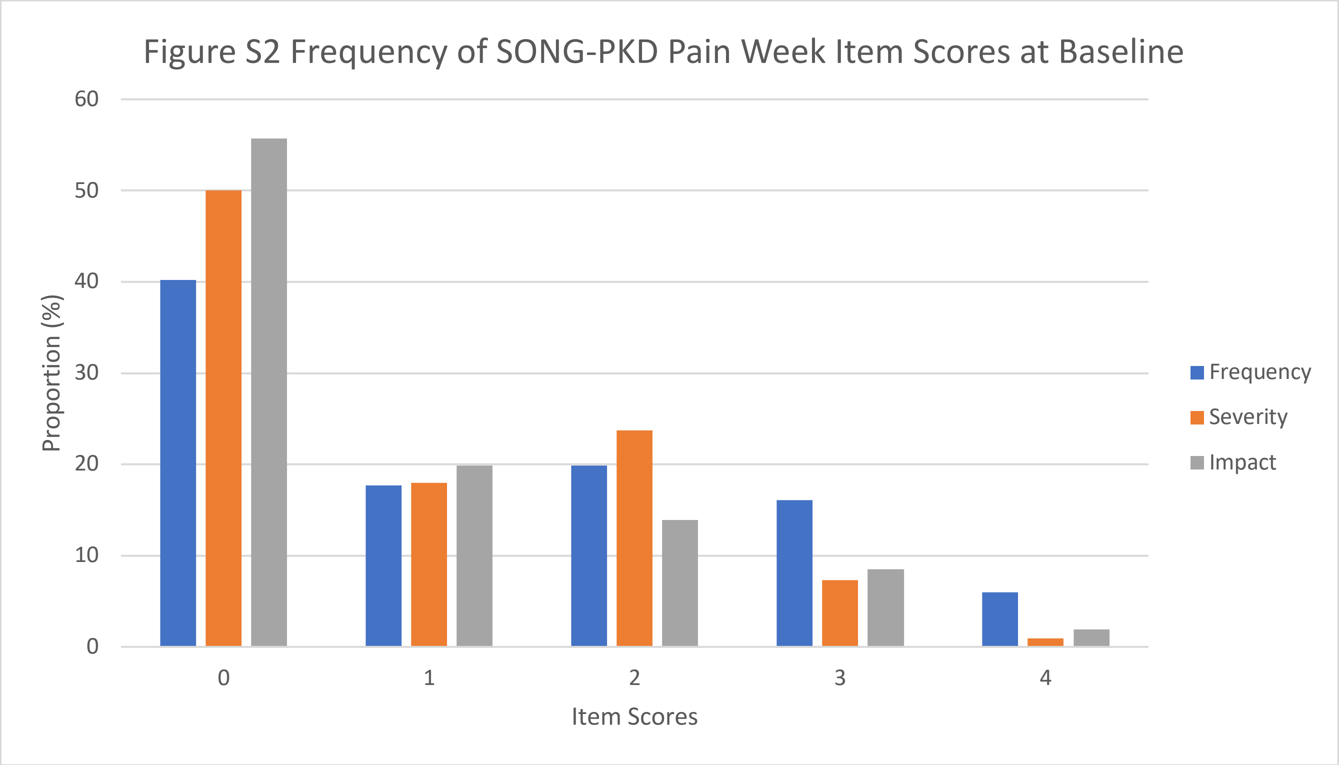
Figure S2 Frequency of SONG-PKD Pain Week Item Scores at Baseline
Proportion (%)
60
50
40
30
20
10
0
0
1
2
3
4
Item Scores
Frequency
Severity
Impact

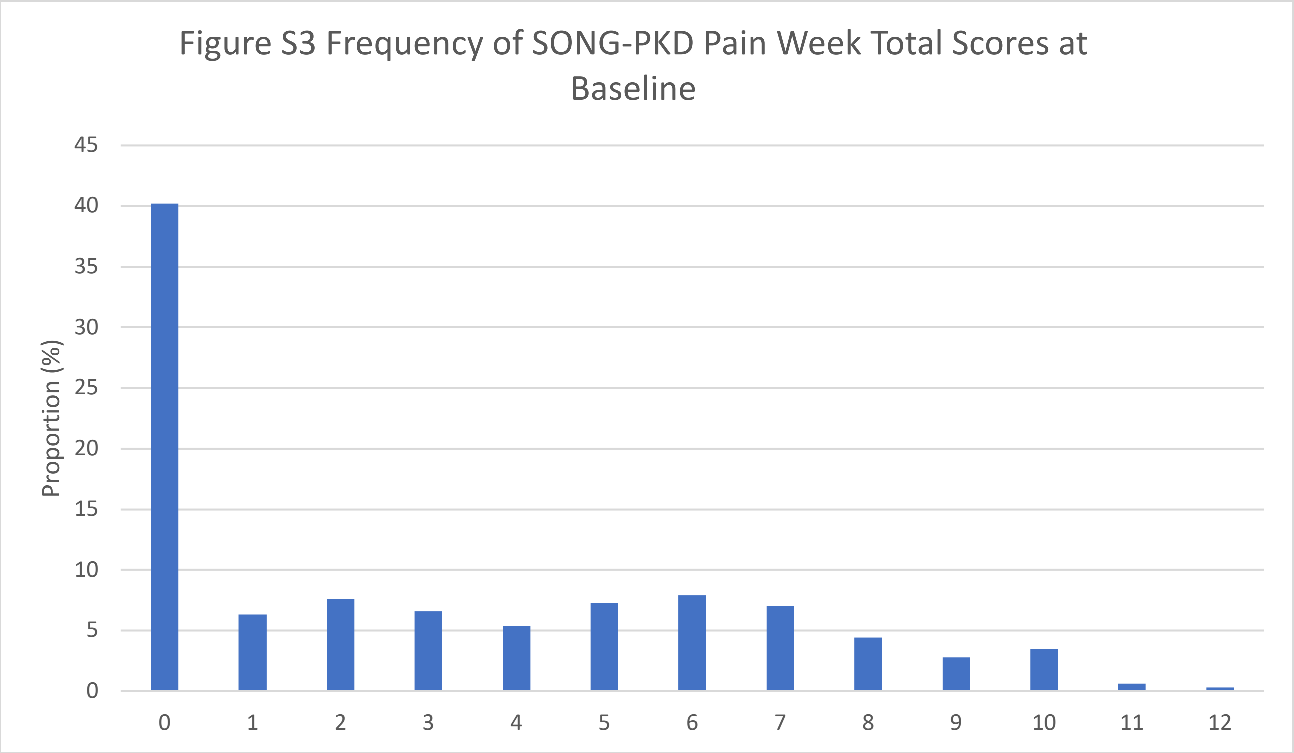
Figure S3 Frequency of SONG-PKD Pain Week Total Scores at Baseline
Proportion (%)
45
40
35
30
25
20
15
10
5
0
0
1
2
3
4
5
6
7
8
9
10
11
12

Table S4 COSMIN Reporting guideline for studies on measurement properties of patient reported outcome measures

Version August 2021

Joel J Gagnier, Jianyu Lai, Lidwine B Mokkink, Caroline B Terwee. COSMIN reporting guideline for studies on measurement properties of patient-reported outcome measures. Qual Life Res. 2021 Aug; 30(8):2197-2218. doi: 10.1007/s11136-021-02822-4

**General Reporting recommendations relevant for all studies on measurement properties**			
**Item Number**	**Item Name**	**Item Description**	
**Report section: Title**			
T1	Patient Reported Outcome Measure (PROM)	The name of the PROM instrument(s) (and version if relevant) being studied	Title page, pg 1
T2	Measurement Property (MP)	What MPs are being studied or more generally, that MPs are being studied (if there are many properties being investigated, for example)	The title indicates that the manuscript describes a validation study. Specific measurement properties are not listed in the title due to space
T3	Study sample	General description of relevant study sample characteristics (e.g., condition of interest, language) and also any intervention or exposure (e.g., treatments) if applicable.	Not specified in the title due to space
**Report section: Abstract**			
A1	PROM	The name of the PROM instrument(s) (and version if relevant) being studied (i.e. the SF-36 or SF-12; language version) or if it concerns an item bank (e.g., PROMIS instruments). The type of	Pg 5 “This study aimed to validate the Standardized Outcomes in Nephrology-PKD (SONG-PKD) Pain measure, which includes three items related to pain (frequency, severity, and

		instrument (e.g. a self reported questionnaire or interview).	impact on life participation) measured on a 5-point Likert scale, in adults with ADPKD”
A2	Measurement Property	What MPs are being studied or more generally, that MPs are being studied (if there are many properties being investigated, for example)	Pg 5 (see response to A5)
A3	Design	The type of study being used to test the properties (e.g., test-retest design, longitudinal study, cohort, cross sectional, case series, randomized etc.). Other details of the study design if relevant (intervention/exposure, description of comparison instruments, outcomes other than PROMs).	Pg 5 “316 adults with ADPKD from 21 countries participated online. …Participants completed a demographic questionnaire, brief medical history and four pain measures at baseline. The pain measures were re-administered 2 days later.
A4	Sample	Inclusion / exclusion criteria. General description of relevant study sample characteristics (e.g., condition of interest, geographic location, language, other relevant demographic and baseline characteristics)	Pg 5 “316 adults with ADPKD from 21 countries participated online. The median (IQR) age of participants was 56 (44-66) years, 219 (69%) were female and 222 (70%) had a university degree or higher.”
A5	Methods	A brief description of the methods for investigating each MP including statistical analyses	Pg 5 “Internal consistency was evaluated with Cronbach’s alpha. Test-retest reliability was assessed using intraclass correlation coefficient (ICC) and convergent validity was assessed using Spearman’s rho. Known groups comparisons for patients with or without a history of kidney complications were performed using a Mann-Whitney rank sum test.”

A6	Results	The main results for all MPs investigated reporting statistics for each result with measures of precision where appropriate.	Pg 5 "The SONG-PKD Pain measure demonstrated high internal consistency (0.94, 95% confidence intervals [CI] 0.93 to 0.95) and test-retest reliability (0.92, 95% CI 0.90 to 0.94). There was high convergence of SONG-PKD Pain with the brief pain inventory short form (BPI-SF; 0.84, 95% CI 0.80 to 0.87) and a visual analogue scale (VAS; 0.84, 95% CI 0.81 to 0.87). There was a significant difference in the median scores of patients with and without a history of complications (4.0 vs 0.0, P<0.001)."
A7	Discussion/Conclusions	A brief description of the results in the context of existing evidence, main strengths and drawbacks and the need for future research on the PROM(s) investigated.	Not addressed in the abstract due to word limit
**Report section: Introduction**			
I1	Name and describe the PROM of interest	Specify the name, type, language, and version of the PROM being investigated and how it was developed. Describe the construct the PROM aims to measure and its subscales; describe the structure of the PROM (e.g., the number of factors, the number of items, scoring algorithm); describe relevant instructions (like time period), and number or type of response categories. State whether the PROM is based on a reflective or formative model.	Pg 8 paragraph 1 "The SONG-PKD Pain measure was developed with the input of patients, caregivers, and health professionals, in accordance with the Consensus-based standards for the selection of health Measurement Instruments (COSMIN) guidelines. The process included a systematic review of PROMs previously used to report pain in ADPKD trials, followed by a multistakeholder workshop to elicit preferences for the most relevant dimensions of pain to include in a pain measure for ADPKD6. … none of the previously used measures captured

		Note: This information may also appear in the methods section in greater detail.	all the dimensions of pain considered most relevant by the workshop delegates, namely its severity, frequency, and its impact on life participation6. The SONG-PKD Pain measure was developed based on these recommendations."<br><br>Further details are provided in the methods section
I2	Target population	Describe the specific target population that the PROM was designed for. The authors need to provide the appropriate and necessary characteristics of this population.	Introduction pg 8 paragraph 1
I3	Citation for the original development of the PROM	The citation for the original development paper(s) should be provided and other highly relevant citations related to the quality of the specific PROM under investigation.	Pg 20, References 3-6:<br><br>3. Cho Y, Rangan G, Logeman C, et al. Am J Kidney Dis. 2020;76(3):361-373. doi:10.1053/j.ajkd.2020.01.005<br>4. Cho Y, Sautenet B, Gutman T, et al 2019;24(12):1214-1224. doi:10.1111/nep.13566<br>5. Cho Y, Tong A, Craig JC, et al. Am J Kidney Dis. 2021;77(2):255-263. doi:10.1053/j.ajkd.2020.05.024

			6. Natale P, Perrone RD, Tong A, et al. Clin Kidney J. 2022;15(3):407-416. doi:10.1093/ckj/sfab110
I4	State of Knowledge & Rationale	A description of the current scientific knowledge (what is known) regarding the MPs of? the PROM under investigation. The authors should provide a literature review or refer to a recent review of all existing evidence of the specific version (e.g., language, short form) of the PROM and explain why the new study is necessary and important. The rational for the current proposed study should be given.	There is a lack of core outcome measure for pain, an outcome of critical importance for patients with autosomal polycystic kidney disease. The SONG-PKD Pain group has developed a novel SONG-PKD Pain measure, a short instrument, capturing aspects of pain prioritized by patients with ADPKD, with plan for implementation in all future trials targeting patients with ADPKD. This is the first report regarding MPs of the SONG-PKD Pain PROM.
I5	Definitions	Specialized terms should be defined or explained.	Acronyms are defined on their first usage. Description of psychometric properties are given in the results section "Data analysis" pg 11-12 e.g.<br><br>"Test-retest stability: The reproducibility of scores between timepoints one and two…."<br><br>"Internal consistency: The correlation of individual item scores within the same instrument…"<br><br>"Convergent validity…the degree to which the scores of one instrument correlate with the scores of other instruments intended to measure the same construct…"

I6	Objectives and Hypotheses	State the specific objective(s) of the research and hypotheses related to the specific PROM under investigation.	Pg 8, paragraph 2 "The COSMIN checklist recommends the assessment of a prescribed set of psychometric properties to establish the validity of PROMs for the condition and population they are intended to measure19. In this study, we aimed to examine the reliability (stability and internal consistency) and construct validity (convergent validity) properties of the SONG-PKD Pain measure, in accordance with the COSMIN recommendations."
**Report section: General Methods**			
GM1	Study Design	State the key elements of the study design	Please see responses below
GM2	Participants	State how the participants were chosen; the inclusion and exclusion criteria. (e.g., if a PROM for a specific condition, then the eligibility and selection criteria should reflect this).	Pg 9 paragraph 1 "Participants were eligible to participate if they were aged 18 years or older, able to read and write in English, and able to provide informed consent. Invitations were sent by email to the Standardized Outcomes in Nephrology (SONG) database. Invitations were also distributed through patient organizations (e.g. PKD Australia and PKD International)."
GM3	PROM administration	An explicit description of how and when the PROM(s) were administered (e.g., in what setting) including data collection devices/system used (e.g. paper based, electronic administration / ePRO) should be provided.	Pg 11 paragraph 3 "At baseline (time point 1), each participant gave informed consent and completed a demographic questionnaire, a brief medical history, and the four pain measures outlined above: SONG-PKD Pain Week, SONG-PKD Pain 24, BPI-SF and VAS. Participants were invited to complete the pain measures again after two days (time point 2), and a second reminder

			was sent after an additional 2 days. A short time period between timepoints 1 and 2 was chosen to minimise confounding of the data arising from significant changes in patient's pain symptoms between administrations of the surveys. The surveys were administered online using Research Electronic Data Capture (REDCap), a secure web-based application for building and managing surveys. The survey was open from August 2022 to November 2022.
GM4	Data collection procedures	Provide information about other data collection, exposure methods (e.g., allocation to interventions) and time points / follow-up points.	See response to GM3
GM5	Power/sample size calculation	Provide a power calculation for all MP analyses. Alternatively, if a rule of thumb is used, state it and the source/citation.	Pg 9 "We estimated a sample size of 450 participants to allow the estimation of the ICC statistics with a 95% confidence interval precision of 0.05, assuming an expected ICC of 0.7 and 10% of missing data in one of the observations. If the observed intraclass correlation coefficient (ICC) is higher than 0.7, the confidence interval width will be smaller. A similar precision is achieved for the Cronbach's alpha estimate, for an expected value of 0.7."
GM6	Statistical analyses	Statistical analyses and tests corresponding to all hypotheses or objectives for all MPs should be reported. Where appropriate, a cut-off for statistical significance should be reported (e.g., p-value less than 0.05). A description of all statistics	Data analyses is described from pages 11-13

		to be used to estimate the magnitude and direction of effect should also be reported, together with measures of variability or precision. Report statistical package used.	
GM7	Missing data	State approaches or plan for dealing with missing data.	Described in the results, Pg 14, paragraph 1 “Participants with missing data were removed prior to data analysis.”
GM8	Post hoc analysis	The report should specify analyses that used data after the data collection period concluded (i.e., if the analyses were post hoc; secondary data analyses) and describe the rationale for any post hoc analyses.	n/a
**Report section: General Results**			
GR1	Missing data	The amount and reasons for missing data should be explained for all analyses for all PROMs (or other outcome measurement instruments) and relevant groups.	Described in the results, Pg 14, paragraph 1 “The reasons for missing data cannot be ascertained as the survey was administered online.”
GR2	Participant/patient Characteristics	The study patients’ characteristics should be described, including baseline PROM scores.	Table 1 patient characteristics<br>Table 2 baseline PROM scores
GR3	Sample size	If one study contained analyses using different sample sizes, the authors should report the sample size for each analysis.	Fig. 2 reports sample size for each analysis
**Report section: Discussion**			
D1	MP evidence	Per measurement property the authors should compare the result to the criteria for good	Pg 16 paragraph 2 “SONG-PKD Pain measure demonstrated acceptable internal consistency,

		measurement properties (e.g., COSMIN criteria)[27], and determine if the specific MP is sufficient or not. Note: This information may also appear in the results section in greater detail in a table for example.	test-retest reliability, and high item-level and domain-level convergence with the BPI-SF and VAS."<br><br>Greater detail is provided in the results
D2	Practical relevance	The authors need to discuss the practical relevance of the findings.	Pgg 18 paragraph 2 "The use of the instrument across trials of ADPKD can provide a standardized measure of pain that is relevant and meaningful to patients. This will contribute to an evidence-base in which patients, caregivers and health professionals can compare and consider the effect of interventions on pain for better decision-making and outcomes for patients."
D3	Strengths and limitations	Strengths and limitations of the study should be discussed. For example, discuss if there were any significant potential biases in the study that could have impacted the results.	Pg 17 paragraph 3 "There are some potential limitations to this study, including the generalizability of the results to populations not captured within the study cohort. The surveys were administered in English and online, which excluded the participation of non-English speakers and those without access to online platforms or with low digital literacy. Most of the participants were from high income countries and had high educational attainment. Further work is needed to assess the psychometric robustness of the measure in other populations, and to determine cultural and language validity."

D4	Generalizability	Generalizability issues related to the PROM results should be discussed. For example, discuss if the results could be generalized to other populations given the sample studied.	See response to D3
D5	Instrument changes	Discuss the need for modifications to the existing PROM or new PROM development. If you conclude that one of the measurement properties is insufficient, you could suggest some modification, or if it is really poor, you could suggest stopping use of the PROM (in the specific population or in general).	n/a, the SONG measure performed well on all psychometric properties tested
D6	Future Research	Report specifically the type of research needed to answer new questions arising out of these findings for the particular MP and PROM investigated.	Pg 18 paragraph 1 "Our exploratory known-group analyses suggests that the SONG-PKD Pain instrument has the potential to distinguish between patients with and without a history of cyst-related complications, including patients with a very recent complication. However, further work is required to conclusively establish known-group validity for this measure. Other psychometric properties such as responsiveness and minimal clinically significant difference are also yet to be determined."
**Report section: Conclusions**			
C1	Conclusions	State the overall conclusions for each MP and of the use PROM investigated.	Pg 18 paragraph 2 "This initial evidence to support validity and reliability of the SONG PKD Pain instrument indicated that it is an appropriate patient-reported outcome measure for patients with ADPKD. The use of the instrument across

			trials of ADPKD can provide a standardized measure of pain that is relevant and meaningful to patients. This will contribute to an evidence-base in which patients, caregivers and health professionals can compare and consider the effect of interventions on pain for better decision-making and outcomes for patients.”
**Report section: Other information**			
O1	Conflict of Interest	State any relevant conflict of interest related to the PROM under investigation (e.g., an author being the PROM developer, funding body etc).	Pg 18 Disclosure statement<br><br>Introduction pg 8 paragraph 1 clearly indicates that the SONG group has developed the instrument being tested.

**Specific Reporting recommendations for studies on Content Validity**			
**Item Number**	**Item Name**	**Item Description**	
CV1	Relevance	Report if and how patients and/or professionals were asked whether each item is relevant for their experience with the condition	The submitted manuscript describes a validation study of the SONG-PKD Pain core outcome measure – content validity is not the focus of this report. However, the development of the tool included a consensus workshop to elicit stakeholder preferences (CV1, CV2, CV 4, CV 6), which is described in the introduction (pg 8 paragraph 1; Ref 6) and cognitive testing (CV3, CV5, CV7) with patients, which is described in
CV2	Comprehensiveness	Report if and how patients and/or professionals were asked whether all key concepts are included	
CV3	Comprehensibility	Report if and how the comprehensibility of the PROM instructions, items, response options, and recall period was assessed	

CV4	Relevance results	Report if all items were considered relevant for the construct, population, and context of use of interest by patients and/or professionals	the methods (pg 10 paragraph 2). The results of these cognitive tests are included as supplementary material (Table S1).
CV5	Response options and recall period	Report whether the response options and recall period were considered appropriate by patients and/or professionals	
CV6	Comprehensiveness results	Report whether patients and/or professionals considered all key concepts to be included in the PROM	
CV7	Comprehensibility results	Report whether patients understood the PROM instructions, items, and response options as intended	

**Specific Reporting recommendations for studies on Structural Validity**			
**Item Number**	**Item Name**	**Item Description**	
SV1	Factor Analyses: Classical Test Theory (CTT) PROMs	Report details of the methods and results for any exploratory or confirmatory factor analyses. State the rational for any explorative factor analyses (e.g., no clear a priori hypotheses). For CFA, describe and justify the factor structure of tested models. Methods and results for checking of the assumptions should be described, the method of estimation, goodness-of-fit statistics and cut-off points for good model fit, including factor loadings of best-fitting model.	n/a structural validity not assessed in this study

SV2	Item Response Theory (IRT) analyses	Type of IRT/Rasch model should be reported. Also report the method of estimation, methods and results for checking of the assumptions (unidimensionality (see factor analysis), local dependency (e.g., residual correlations), monotonicity; (e.g. Mokken scaling), goodness-of-fit statistics, and cut-off points for goodness of item/model fit, and all item parameters.	n/a structural validity not assessed in this study

**Specific Reporting recommendations for studies on Internal Consistency**			
**Item Number**	**Item Name**	**Item Description**	
IC1	Unit of measurement	Report internal consistency methods and results for each unidimensional scale or subscale. Report all evidence or assumptions associated with unidimensionality.	Methods pg 12 “Internal consistency: The correlation of individual item scores within the same instrument was assessed using Cronbach’s alpha in a complete case analysis at timepoint 1, where values ≥ 0.70 indicated adequate consistency”<br><br>Results Table 2<br><br>Unidimensionality has been assumed as each of the three subscales ask questions specific to pain.

IC2	Continuous scores	Report Cronbach's alpha or omega statistics. Report other statistics calculated for internal consistency of continuous scores.	Table 2
IC3	Dichotomous scores	Report Cronbach's alpha or Kuder-Richardson coefficient. Report other statistics calculated for internal consistency of dichotomous scores.	n/a

**Specific Reporting recommendations for studies on Cross-Cultural Validity\Measurement Invariance**			
**Item Number**	**Item Name**	**Item Description**	
CCV1	Comparator Group(s)	Report characteristics of (sub)groups being compared. Include sample sizes in each group.	n/a Cross-Cultural Validity\Measurement Invariance not assessed in this study
CCV2	Factor Analyses: Classical Test Theory (CTT) PROMs	Report details of the methods and results for multiple-group confirmatory factor analyses, logistic regression analyses, or other analyses performed. Describe and justify the series of tested models, including constraints of factor loadings, intercepts and variances in CFA. Methods and results for checking of the assumptions should be described. criteria to define invariance. Describe the method of estimation, goodness-of-fit statistics and criteria used to flag items for measurement invariance.	n/a Cross-Cultural Validity\Measurement Invariance not assessed in this study
CCV3	Item Response Theory (IRT) analyses	Type of IRT/Rasch model should be reported. Also report the methods and results for checking of the assumptions (unidimensionality (see factor analysis), local dependency (e.g., residual correlations), monotonicity; (e.g. Mokken scaling).. Describe statistical packages, method of estimation, criteria used to flag items for DIF, and methods and results of all model comparisons.	n/a Cross-Cultural Validity\Measurement Invariance not assessed in this study

Specific Reporting recommendations for studies on Reliability			
**Item Number**	**Item Name**	**Item Description**	
R1	PROM Administrations	Report the total number of measurements made and if the measurements were applied to the same samples using the same PROM. The process of administrating the measurements to the patients should be described, including who administered it (i.e., did the patient complete it or was there a proxy), when, how and any time intervals between administrations should be reported. This should include: time interval between repeated measurements (e.g., was the patient stable or not), the test type (e.g. a self-administered questionnaire, an interview-based PROM), the setting in which the instrument was administered (e.g., at the hospital, or at home), and the instructions given for completing it. If relevant, other instruments or measurements accompanying the repeated PROM measurement. Also, if relevant, the independence (whether the PROM was completed without knowledge of the previous scores) of the administrations.	Methods pg 9: Selection and recruitment of participants<br>Methods pg 11: Data collection
R2	Statistical analyses	All statistical analyses and results specific to the reliability assessment(s) should be described and their use justified (e.g., the intraclass correlation coefficient (ICC) model or type of Kappa coefficient used). Also, describe the variance components, and the weighting scheme used for ordinal scores (e.g., linear or quadratic weights).	Methods pg 11-13: Data Analyses

R3	Methods to improve reliability	Report any methods used to improve reliability such as restriction of the sample, training of researchers and standardization of methods, and averaging of repeated measurements.	n/a

**Specific Reporting recommendations for studies on Measurement Error**			
**Item Number**	**Item Name**	**Item Description**	
ME1	PROM administrations	Report the total number of measurements made and if the measurements were applied to the same samples using the same PROM. The process of administrating the measurements to the patients should be described, including who administered it (i.e., did the patient complete it or was there a proxy), when, how and any time intervals between administrations should be reported. This should include: time interval between repeated measurements (e.g., was the patient stable or not), the test type (e.g. a self-administered questionnaire, an interview-based PROM), the setting in which the instrument was administered (e.g., at the hospital, or at home), and the instructions given for completing it. If relevant, other instruments or measurements accompanying the repeated PROM measurement. Also, if relevant, the independence (whether the PROM was completed without knowledge of the previous completion) of the administrations.	n/a measurement error not assessed in this study

ME2	Statistical analyses	All statistical analyses and results specific to measurement error assessment(s) should be described and their use justified. Specifically, for continuous scores report the Standard Error of Measurement (SEM; Specify the exact model used to calculate the SEM (i.e., SEM consistency or SEM agreement)), Smallest Detectable Change (SDC; specify formula used, included the model of the SEM when based on the SEM) or Limits of Agreement (LoA). For dichotomous/nominal/ordinal scores report marginals (raw data) and the percentage specific (e.g. positive and negative) agreement.	n/a measurement error not assessed in this study

Specific Reporting recommendations for studies on Criterion Validity			
**Item Number**	**Item Name**	**Item Description**	
CriV1	Criterion	Report the details of the criterion used and why it was used. Justification of the gold standard must be reported. Also, describe (if applicable) how and why the criterion was dichotomized or classified. Also, how and when the criterion was administered (e.g., if independent from the PROM).	n/a criterion validity not assessed in this study
CriV2	Continuous scores	Report correlations (when criterion has continuous scores) or the area under the receiver operating characteristic (ROC) curve (when criterion is dichotomous).	n/a criterion validity not assessed in this study
CriV3	Categorical scores	Described how (and why) the PROM was dichotomized or made into multiple categories. Report sensitivity and specificity statistics.	n/a criterion validity not assessed in this study

Specific Reporting recommendations for studies on Hypotheses Testing for Construct Validity			
**Item Number**	**Item Name**	**Item Description**	
ConV1	Comparator instrument(s)	The comparator instruments should be appropriately described in terms of the construct(s) they intend to measure. Report the measurement properties of the comparator instruments and related citations or data.	Methods pg 10-11

ConV2	Comparator Group(s)	Report characteristics of groups being compared. Include sample sizes in each group.	Patient characteristics and sample size are provided in Table 1<br><br>A subgroup analysis of convergent validity was performed and sample size is reported in the results (pg 15). Characteristics of this subgroup of patients is not reported
ConV3	Hypotheses	Report all hypotheses including the direction and magnitude of the expected correlations between the PROM of interest and another measurement instrument, or the direction and magnitude of differences in scores of the PROM between groups.	Not reported
ConV4	Statistical analyses	Report all statistical methods and results used to test each hypothesis.	
ConV5	Results	Report which specific results are in accordance with its hypothesis.	

**Specific Reporting recommendations for studies on Responsiveness**			
**Item Number**	**Item Name**	**Item Description**	
Resp1	Comparison Instrument(s)	The comparator instruments should be appropriately described in terms of the construct(s) they intend to measure. Report the	n/a responsiveness not assessed in this study

		measurement properties of the comparator instruments and related citations or data.	
Resp2	Comparator Group(s)	Report characteristics of groups being compared. Include sample sizes in each group.	n/a responsiveness not assessed in this study
Resp3	Hypotheses	Report all hypotheses including the direction and magnitude of the expected correlations between changes in the PROM of interest and change in another measurement instrument, or the direction and magnitude of differences in change scores of the PROM between groups.	n/a responsiveness not assessed in this study
Resp4	Measurement procedures	Report if measurements were applied to the same sample using the same instruments. Describe the measurement procedures, including time intervals between different measurement instruments.	n/a responsiveness not assessed in this study
Resp5	Interim period	The interim period between time points should be described.	n/a responsiveness not assessed in this study
Resp6	Intervention/Exposure	Describe the intervention given or exposure in the interim period if relevant.	n/a responsiveness not assessed in this study
Resp7	Patients changed	Report the proportion of patients that improved or deteriorated (and the details of any anchor used) on the construct measured on all PROMs. Report any changes in scores of the PROM in the target population for the research application relative to the predefined hypotheses	n/a responsiveness not assessed in this study

Resp8	Statistical analyses	Report all statistical methods and results used to test each hypothesis.	n/a responsiveness not assessed in this study
Resp9	Results	Report which specific results are in accordance with its hypothesis.	n/a responsiveness not assessed in this study
